# 1-[2-(Trit­yloxy)phen­yl]ethanone

**DOI:** 10.1107/S1600536813012956

**Published:** 2013-05-18

**Authors:** Pengying Zhao

**Affiliations:** a Department of Basic Science, Tianjin Agricultural University, Tianjin 300384, People’s Republic of China

## Abstract

In the title compound, C_27_H_22_O_2_, the acetyl group is nearly coplanar with the the ring to which it attacted [O—C—C—C torsion angle = −5.5 (3)°]. The three phenyl groups of the tri­phenyl­methyl substituent are mutually nearly perpendicular, making dihedral angles of 89.87 (11) and 78.29 (11) and 60.34 (11)°. Two intra­molecular C—H⋯ O hydrogen bonds occur. In the crystal, C—H⋯ O hydrogen bonds link the moleclues into chains along the *b-*axis direction.

## Related literature

For general background to tri­phenyl­methyl, see: Casanova *et al.* (2006 ▶); Aldaye & Sleiman (2007 ▶).
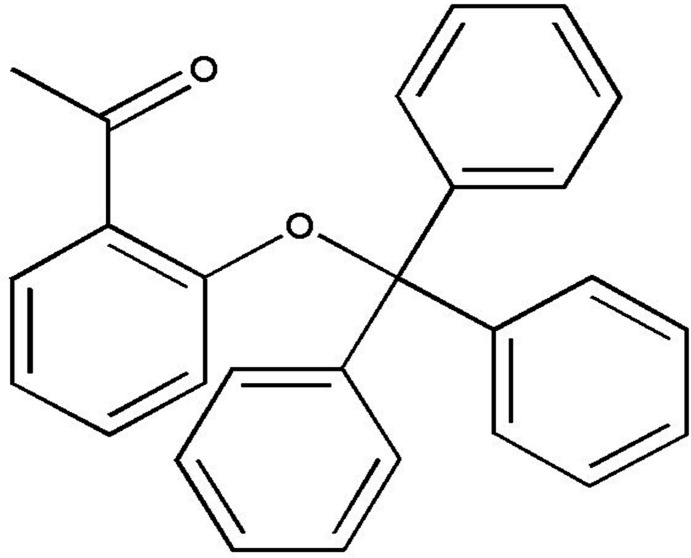


## Experimental

### 

#### Crystal data

C_27_H_22_O_2_
*M* *_r_* = 378.45
Orthorhombic, 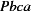

*a* = 15.6996 (17) Å
*b* = 8.8767 (9) Å
*c* = 29.591 (3) Å
*V* = 4123.8 (7) Å^3^
*Z* = 8
Mo *K*α radiation
μ = 0.08 mm^−1^
*T* = 296 K
0.26 × 0.24 × 0.20 mm

#### Data collection

Bruker SMART CCD area-detector diffractometer
Absorption correction: multi-scan (*SADABS*; Bruker, 1996 ▶) *T*_min_ = 0.674, *T*_max_ = 0.745
21669 measured reflections
3653 independent reflections
2496 reflections with *I* > 2σ(*I*)
*R*_int_ = 0.053

#### Refinement

*R*[*F* ^2^ > 2σ(*F* ^2^)] = 0.045
*wR*(*F* ^2^) = 0.128
*S* = 1.02
3653 reflections
263 parameters
H-atom parameters constrained
Δρ_max_ = 0.13 e Å^−3^
Δρ_min_ = −0.16 e Å^−3^

### 

Data collection: *SMART* (Bruker, 2007 ▶); cell refinement: *SAINT* (Bruker, 2007 ▶); data reduction: *SAINT*; program(s) used to solve structure: *SHELXS97* (Sheldrick, 2008 ▶); program(s) used to refine structure: *SHELXL97* (Sheldrick, 2008 ▶); molecular graphics: *DIAMOND* (Brandenburg, 1999 ▶); software used to prepare material for publication: *SHELXTL* (Sheldrick, 2008 ▶).

## Supplementary Material

Crystal structure: contains datablock(s) I, global. DOI: 10.1107/S1600536813012956/ds2228sup1.cif

Structure factors: contains datablock(s) I. DOI: 10.1107/S1600536813012956/ds2228Isup2.hkl

Supplementary material file. DOI: 10.1107/S1600536813012956/ds2228Isup3.cml

Additional supplementary materials:  crystallographic information; 3D view; checkCIF report

## Figures and Tables

**Table 1:** Hydrogen-bond geometry (Å, °)

*D*—H⋯*A*	*D*—H	H⋯*A*	*D*⋯*A*	*D*—H⋯*A*
C14—H14⋯O1^i^	0.93	2.56	3.444 (3)	158
C21—H21⋯O2	0.93	2.46	2.810 (3)	103
C5—H5⋯O1	0.93	2.36	2.701 (3)	101

Symmetry code: (i) 
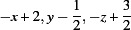
.

**Fig. 1.**
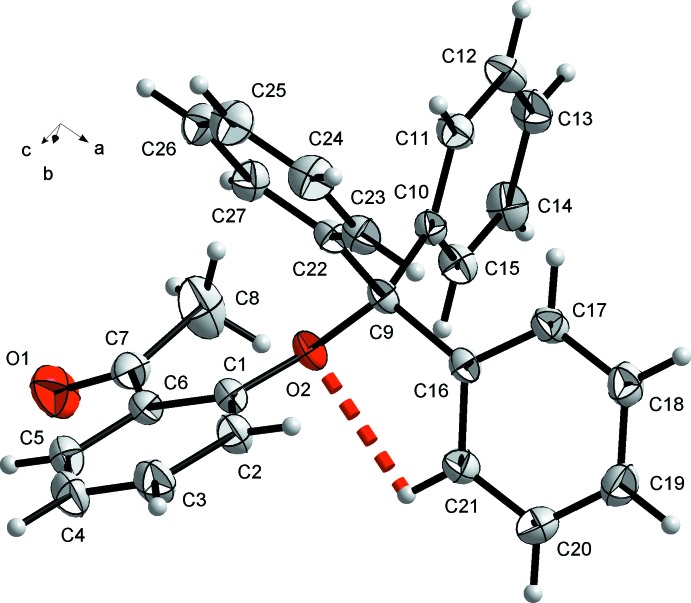
A view of the molecular structure of the title compound with the intramolecular hydrogen bond. The displacement ellipsoids are drawn at the 30% probability level.

**Fig. 2.**
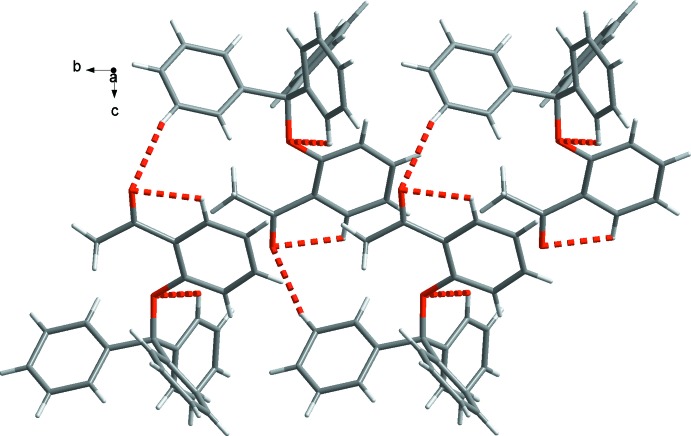
A view of the hydrogen bonded polymeric chain. The hydrogen bonds are shown as dashed lines.

## supplementary crystallographic information

### Comment

Trityloxy aromatic ketones are useful intermediates in organic synthesis (Casanova *et al.* 2006). The X-ray analysis of the title compound C_27_H_22_O_2_ (Fig. 1) is shown in Fig. 1. The ethanoyl group is nearly coplanar with the the ring to which it attacted, reflected by the O1—C7—C6—C5 torsion angle of -5.4 (3)°. Phenyl ring (C10—C15) makes dihedral angles of 89.87 (11) and 78.29 (11)° with the phenyl rings (C16—C21) and (C22—C27). The intermolecular hydrogen bonds C14—H14···O1 and C21—H21···O2 link adjacent moleclues, forming an infinite one-dimensional chain along the *b* axis. Furthermore, each molecule of the asymmetric unit exhibits an intramolecular C5—H5···O1 hydrogen bond.

### Experimental

A 50 ml flask, fitted with a condenser, was charged with *o*-hydroxyacetophenone (3.00 g, 22.03 mmol), trityl chloride (5.84 g, 20.95 mmol), dry CH_2_Cl_2_ (20 ml), triethylamine (3.1 ml, 22.24 mmol) and 4-dimethylaminopyridine (270 mg). The reaction was refluxed for 96 h under nitrogen. After cooling to room temperature, the reaction mixture was washed three times with 10% aqueous NaOH, dried with anhydrous K_2_CO_3_. The crude material was purified by column chromatography(5% anhydrous Na_2_CO_3_ in silica gel, hexane:ethyl acetate: NEt_3_= 100:10:1 as eluent) to provide 6.58 g of *o*-trityloxyacetophenone as a white solid (83% yield). Crystallization from EtOH (containing 1% NEt_3_) afforded the single-crystal.

### Refinement

H atoms bonded to C were positioned with idealized geometry using a riding model with the aromatic C—H = 0.93 Å, methyl C—H = 0.96 Å. All H atoms were refined with isotropic displacement parameters set at 1.2 *U*_eq_(C-aromati) and 1.5 *U*_eq_ (*C*-methyl) of the parent atom.

### Crystal data

**Table:**

C_27_H_22_O_2_	*F*(000) = 1600
*M**_r_* = 378.45	*D*_x_ = 1.219 Mg m^−^^3^
Orthorhombic, *P**b**c**a*	Mo *K*α radiation, λ = 0.71073 Å
Hall symbol: -P 2ac 2ab	Cell parameters from 3063 reflections
*a* = 15.6996 (17) Å	θ = 2.6–21.8°
*b* = 8.8767 (9) Å	µ = 0.08 mm^−^^1^
*c* = 29.591 (3) Å	*T* = 296 K
*V* = 4123.8 (7) Å^3^	Block, colourless
*Z* = 8	0.26 × 0.24 × 0.20 mm

### Data collection

**Table:**

Bruker SMART CCD area-detector diffractometer	3653 independent reflections
Radiation source: fine-focus sealed tube	2496 reflections with *I* > 2σ(*I*)
Graphite monochromator	*R*_int_ = 0.053
phi and ω scans	θ_max_ = 25.0°, θ_min_ = 1.4°
Absorption correction: multi-scan (*SADABS*; Bruker, 1996)	*h* = −18→15
*T*_min_ = 0.674, *T*_max_ = 0.745	*k* = −10→10
21669 measured reflections	*l* = −35→28

### Refinement

**Table:**

Refinement on *F*^2^	Primary atom site location: structure-invariant direct methods
Least-squares matrix: full	Secondary atom site location: difference Fourier map
*R*[*F*^2^ > 2σ(*F*^2^)] = 0.045	Hydrogen site location: inferred from neighbouring sites
*wR*(*F*^2^) = 0.128	H-atom parameters constrained
*S* = 1.02	*w* = 1/[σ^2^(*F*_o_^2^) + (0.0619*P*)^2^ + 0.751*P*] where *P* = (*F*_o_^2^ + 2*F*_c_^2^)/3
3653 reflections	(Δ/σ)_max_ = 0.001
263 parameters	Δρ_max_ = 0.13 e Å^−^^3^
0 restraints	Δρ_min_ = −0.16 e Å^−^^3^

### Special details

**Table:**

Geometry. All e.s.d.'s (except the e.s.d. in the dihedral angle between two l.s. planes) are estimated using the full covariance matrix. The cell e.s.d.'s are taken into account individually in the estimation of e.s.d.'s in distances, angles and torsion angles; correlations between e.s.d.'s in cell parameters are only used when they are defined by crystal symmetry. An approximate (isotropic) treatment of cell e.s.d.'s is used for estimating e.s.d.'s involving l.s. planes.
Refinement. Refinement of *F^2^* against ALL reflections. The weighted *R*-factor *wR* and goodness of fit *S* are based on *F^2^*, conventional *R*-factors *R* are based on *F*, with *F* set to zero for negative *F^2^*. The threshold expression of *F^2^* > σ(*F^2^*) is used only for calculating *R*-factors(gt) *etc*. and is not relevant to the choice of reflections for refinement. *R*-factors based on *F^2^* are statistically about twice as large as those based on *F*, and *R*- factors based on ALL data will be even larger.

### Fractional atomic coordinates and isotropic or equivalent isotropic displacement parameters (Å^2^)

**Table:**

	*x*	*y*	*z*	*U*_iso_*/*U*_eq_	
O1	0.83938 (14)	0.9201 (2)	0.78288 (6)	0.0885 (6)	
O2	0.97143 (9)	0.99213 (14)	0.66452 (4)	0.0430 (4)	
C1	0.93023 (12)	1.1103 (2)	0.68509 (6)	0.0418 (5)	
C2	0.93469 (14)	1.2572 (2)	0.66877 (7)	0.0518 (6)	
H2	0.9646	1.2774	0.6423	0.062*	
C3	0.89509 (16)	1.3726 (3)	0.69158 (8)	0.0652 (7)	
H3	0.8979	1.4703	0.6803	0.078*	
C4	0.85108 (17)	1.3441 (3)	0.73112 (9)	0.0724 (8)	
H4	0.8248	1.4222	0.7467	0.087*	
C5	0.84667 (15)	1.1997 (3)	0.74704 (8)	0.0626 (7)	
H5	0.8164	1.1812	0.7735	0.075*	
C6	0.88591 (13)	1.0789 (2)	0.72507 (6)	0.0456 (5)	
C7	0.87505 (15)	0.9273 (3)	0.74669 (7)	0.0537 (6)	
C8	0.9036 (2)	0.7862 (3)	0.72541 (9)	0.0893 (10)	
H8A	0.8850	0.7021	0.7432	0.134*	
H8B	0.9647	0.7857	0.7235	0.134*	
H8C	0.8799	0.7788	0.6956	0.134*	
C9	0.98726 (12)	0.9888 (2)	0.61572 (6)	0.0372 (5)	
C16	1.07153 (12)	1.0672 (2)	0.60443 (6)	0.0371 (4)	
C21	1.11903 (13)	1.1444 (2)	0.63622 (7)	0.0488 (5)	
H21	1.0994	1.1511	0.6658	0.059*	
C20	1.19551 (14)	1.2119 (3)	0.62452 (8)	0.0605 (6)	
H20	1.2261	1.2654	0.6461	0.073*	
C19	1.22651 (15)	1.2004 (3)	0.58119 (8)	0.0604 (6)	
H19	1.2777	1.2462	0.5734	0.073*	
C18	1.18105 (15)	1.1206 (3)	0.54957 (8)	0.0556 (6)	
H18	1.2020	1.1112	0.5203	0.067*	
C17	1.10481 (13)	1.0543 (2)	0.56092 (7)	0.0462 (5)	
H17	1.0749	1.0001	0.5392	0.055*	
C22	0.90861 (12)	1.0482 (2)	0.59090 (6)	0.0396 (5)	
C23	0.91211 (14)	1.1540 (2)	0.55649 (7)	0.0481 (5)	
H23	0.9644	1.1939	0.5478	0.058*	
C24	0.83844 (16)	1.2008 (3)	0.53496 (8)	0.0608 (6)	
H24	0.8417	1.2712	0.5118	0.073*	
C25	0.76079 (16)	1.1442 (3)	0.54752 (8)	0.0662 (7)	
H25	0.7116	1.1761	0.5329	0.079*	
C26	0.75589 (15)	1.0406 (3)	0.58172 (8)	0.0605 (6)	
H26	0.7032	1.0027	0.5906	0.073*	
C27	0.82935 (14)	0.9922 (2)	0.60313 (7)	0.0514 (5)	
H27	0.8255	0.9209	0.6261	0.062*	
C10	1.00059 (12)	0.8202 (2)	0.60568 (6)	0.0373 (5)	
C11	0.96804 (14)	0.7533 (2)	0.56714 (7)	0.0488 (5)	
H11	0.9331	0.8082	0.5479	0.059*	
C12	0.98735 (15)	0.6048 (2)	0.55720 (8)	0.0612 (6)	
H12	0.9649	0.5604	0.5313	0.073*	
C13	1.03894 (16)	0.5228 (2)	0.58499 (8)	0.0610 (6)	
H13	1.0520	0.4234	0.5779	0.073*	
C14	1.07137 (16)	0.5871 (2)	0.62336 (8)	0.0586 (6)	
H14	1.1060	0.5311	0.6425	0.070*	
C15	1.05261 (14)	0.7353 (2)	0.63351 (7)	0.0487 (5)	
H15	1.0753	0.7786	0.6595	0.058*	

### Atomic displacement parameters (Å^2^)

**Table:**

	*U*^11^	*U*^22^	*U*^33^	*U*^12^	*U*^13^	*U*^23^
O1	0.1163 (16)	0.1011 (14)	0.0480 (10)	−0.0199 (12)	0.0248 (10)	0.0035 (9)
O2	0.0546 (9)	0.0418 (7)	0.0326 (7)	0.0061 (7)	0.0067 (6)	−0.0006 (6)
C1	0.0427 (11)	0.0451 (12)	0.0376 (11)	0.0000 (9)	0.0046 (9)	−0.0088 (9)
C2	0.0614 (15)	0.0440 (12)	0.0498 (13)	−0.0001 (11)	0.0126 (11)	−0.0050 (10)
C3	0.0753 (17)	0.0466 (13)	0.0737 (17)	0.0049 (12)	0.0114 (14)	−0.0114 (12)
C4	0.0726 (18)	0.0660 (18)	0.0786 (18)	0.0063 (14)	0.0219 (14)	−0.0260 (14)
C5	0.0603 (15)	0.0739 (17)	0.0536 (14)	−0.0059 (13)	0.0174 (12)	−0.0186 (13)
C6	0.0415 (12)	0.0563 (13)	0.0389 (11)	−0.0042 (10)	0.0022 (9)	−0.0078 (10)
C7	0.0524 (13)	0.0711 (15)	0.0378 (12)	−0.0140 (12)	0.0019 (10)	0.0015 (11)
C8	0.132 (3)	0.0568 (16)	0.0792 (19)	0.0034 (17)	0.0404 (18)	0.0150 (14)
C9	0.0459 (12)	0.0379 (10)	0.0278 (10)	0.0026 (9)	0.0039 (8)	0.0004 (8)
C16	0.0413 (11)	0.0351 (10)	0.0348 (10)	0.0042 (9)	0.0012 (9)	0.0005 (8)
C21	0.0468 (12)	0.0596 (13)	0.0400 (11)	0.0002 (10)	−0.0012 (10)	−0.0072 (10)
C20	0.0467 (14)	0.0689 (16)	0.0659 (16)	−0.0072 (11)	−0.0061 (12)	−0.0107 (12)
C19	0.0463 (13)	0.0641 (15)	0.0709 (17)	−0.0071 (11)	0.0085 (12)	0.0025 (13)
C18	0.0578 (14)	0.0603 (14)	0.0488 (13)	−0.0042 (12)	0.0143 (11)	0.0048 (11)
C17	0.0541 (13)	0.0460 (12)	0.0386 (11)	−0.0025 (10)	0.0040 (10)	−0.0021 (9)
C22	0.0447 (12)	0.0359 (10)	0.0381 (11)	0.0047 (9)	0.0006 (9)	−0.0042 (8)
C23	0.0542 (13)	0.0427 (12)	0.0474 (12)	0.0051 (10)	−0.0029 (10)	0.0002 (10)
C24	0.0679 (17)	0.0546 (14)	0.0599 (15)	0.0125 (12)	−0.0114 (12)	0.0077 (11)
C25	0.0555 (15)	0.0696 (16)	0.0734 (17)	0.0196 (13)	−0.0160 (13)	−0.0010 (14)
C26	0.0426 (13)	0.0661 (16)	0.0730 (16)	0.0032 (11)	−0.0019 (12)	−0.0067 (13)
C27	0.0497 (13)	0.0503 (12)	0.0543 (13)	0.0022 (10)	0.0039 (11)	0.0006 (10)
C10	0.0396 (11)	0.0353 (10)	0.0369 (11)	0.0006 (9)	0.0058 (9)	0.0003 (8)
C11	0.0516 (13)	0.0473 (12)	0.0475 (12)	0.0041 (10)	−0.0032 (10)	−0.0068 (10)
C12	0.0666 (16)	0.0508 (13)	0.0662 (15)	−0.0003 (12)	0.0013 (12)	−0.0208 (12)
C13	0.0672 (16)	0.0377 (12)	0.0781 (17)	0.0027 (11)	0.0141 (13)	−0.0065 (12)
C14	0.0682 (16)	0.0435 (13)	0.0642 (15)	0.0125 (11)	0.0062 (12)	0.0096 (11)
C15	0.0546 (13)	0.0480 (12)	0.0436 (12)	0.0054 (10)	0.0005 (10)	0.0018 (10)

### Geometric parameters (Å, º)

**Table:**

O1—C7	1.210 (2)	C19—C18	1.374 (3)
O2—C1	1.374 (2)	C19—H19	0.9300
O2—C9	1.466 (2)	C18—C17	1.375 (3)
C1—C2	1.393 (3)	C18—H18	0.9300
C1—C6	1.401 (3)	C17—H17	0.9300
C2—C3	1.375 (3)	C22—C23	1.386 (3)
C2—H2	0.9300	C22—C27	1.388 (3)
C3—C4	1.382 (3)	C23—C24	1.384 (3)
C3—H3	0.9300	C23—H23	0.9300
C4—C5	1.367 (3)	C24—C25	1.370 (3)
C4—H4	0.9300	C24—H24	0.9300
C5—C6	1.397 (3)	C25—C26	1.370 (3)
C5—H5	0.9300	C25—H25	0.9300
C6—C7	1.500 (3)	C26—C27	1.384 (3)
C7—C8	1.472 (3)	C26—H26	0.9300
C8—H8A	0.9600	C27—H27	0.9300
C8—H8B	0.9600	C10—C15	1.383 (3)
C8—H8C	0.9600	C10—C11	1.384 (3)
C9—C22	1.530 (3)	C11—C12	1.384 (3)
C9—C16	1.532 (3)	C11—H11	0.9300
C9—C10	1.540 (3)	C12—C13	1.364 (3)
C16—C21	1.382 (3)	C12—H12	0.9300
C16—C17	1.394 (3)	C13—C14	1.369 (3)
C21—C20	1.386 (3)	C13—H13	0.9300
C21—H21	0.9300	C14—C15	1.381 (3)
C20—C19	1.375 (3)	C14—H14	0.9300
C20—H20	0.9300	C15—H15	0.9300
			
C1—O2—C9	122.10 (14)	C18—C19—H19	120.4
O2—C1—C2	122.52 (17)	C20—C19—H19	120.4
O2—C1—C6	117.15 (17)	C19—C18—C17	120.4 (2)
C2—C1—C6	120.27 (18)	C19—C18—H18	119.8
C3—C2—C1	120.3 (2)	C17—C18—H18	119.8
C3—C2—H2	119.8	C18—C17—C16	121.1 (2)
C1—C2—H2	119.8	C18—C17—H17	119.4
C2—C3—C4	120.3 (2)	C16—C17—H17	119.4
C2—C3—H3	119.8	C23—C22—C27	118.02 (19)
C4—C3—H3	119.8	C23—C22—C9	123.64 (18)
C5—C4—C3	119.3 (2)	C27—C22—C9	118.34 (17)
C5—C4—H4	120.4	C24—C23—C22	120.5 (2)
C3—C4—H4	120.4	C24—C23—H23	119.7
C4—C5—C6	122.4 (2)	C22—C23—H23	119.7
C4—C5—H5	118.8	C25—C24—C23	120.6 (2)
C6—C5—H5	118.8	C25—C24—H24	119.7
C5—C6—C1	117.4 (2)	C23—C24—H24	119.7
C5—C6—C7	116.10 (19)	C24—C25—C26	119.8 (2)
C1—C6—C7	126.52 (19)	C24—C25—H25	120.1
O1—C7—C8	118.4 (2)	C26—C25—H25	120.1
O1—C7—C6	118.5 (2)	C25—C26—C27	120.0 (2)
C8—C7—C6	123.11 (19)	C25—C26—H26	120.0
C7—C8—H8A	109.5	C27—C26—H26	120.0
C7—C8—H8B	109.5	C26—C27—C22	121.1 (2)
H8A—C8—H8B	109.5	C26—C27—H27	119.5
C7—C8—H8C	109.5	C22—C27—H27	119.5
H8A—C8—H8C	109.5	C15—C10—C11	118.31 (18)
H8B—C8—H8C	109.5	C15—C10—C9	119.69 (17)
O2—C9—C22	109.22 (15)	C11—C10—C9	121.75 (17)
O2—C9—C16	110.62 (15)	C10—C11—C12	120.2 (2)
C22—C9—C16	115.84 (15)	C10—C11—H11	119.9
O2—C9—C10	103.44 (14)	C12—C11—H11	119.9
C22—C9—C10	110.60 (15)	C13—C12—C11	120.6 (2)
C16—C9—C10	106.39 (14)	C13—C12—H12	119.7
C21—C16—C17	117.84 (19)	C11—C12—H12	119.7
C21—C16—C9	122.88 (17)	C12—C13—C14	119.9 (2)
C17—C16—C9	119.20 (17)	C12—C13—H13	120.1
C16—C21—C20	120.78 (19)	C14—C13—H13	120.1
C16—C21—H21	119.6	C13—C14—C15	119.9 (2)
C20—C21—H21	119.6	C13—C14—H14	120.1
C19—C20—C21	120.5 (2)	C15—C14—H14	120.1
C19—C20—H20	119.7	C14—C15—C10	121.0 (2)
C21—C20—H20	119.7	C14—C15—H15	119.5
C18—C19—C20	119.3 (2)	C10—C15—H15	119.5
			
C9—O2—C1—C2	−30.2 (3)	C19—C18—C17—C16	0.4 (3)
C9—O2—C1—C6	152.75 (17)	C21—C16—C17—C18	−2.1 (3)
O2—C1—C2—C3	−177.4 (2)	C9—C16—C17—C18	−178.78 (18)
C6—C1—C2—C3	−0.5 (3)	O2—C9—C22—C23	132.84 (18)
C1—C2—C3—C4	0.5 (4)	C16—C9—C22—C23	7.2 (3)
C2—C3—C4—C5	−0.6 (4)	C10—C9—C22—C23	−114.0 (2)
C3—C4—C5—C6	0.7 (4)	O2—C9—C22—C27	−47.8 (2)
C4—C5—C6—C1	−0.6 (4)	C16—C9—C22—C27	−173.50 (17)
C4—C5—C6—C7	−179.9 (2)	C10—C9—C22—C27	65.4 (2)
O2—C1—C6—C5	177.59 (18)	C27—C22—C23—C24	−0.5 (3)
C2—C1—C6—C5	0.5 (3)	C9—C22—C23—C24	178.87 (18)
O2—C1—C6—C7	−3.3 (3)	C22—C23—C24—C25	0.5 (3)
C2—C1—C6—C7	179.6 (2)	C23—C24—C25—C26	0.1 (4)
C5—C6—C7—O1	−5.5 (3)	C24—C25—C26—C27	−0.7 (4)
C1—C6—C7—O1	175.4 (2)	C25—C26—C27—C22	0.7 (3)
C5—C6—C7—C8	172.8 (2)	C23—C22—C27—C26	−0.1 (3)
C1—C6—C7—C8	−6.4 (4)	C9—C22—C27—C26	−179.52 (18)
C1—O2—C9—C22	−40.9 (2)	O2—C9—C10—C15	−45.7 (2)
C1—O2—C9—C16	87.8 (2)	C22—C9—C10—C15	−162.49 (17)
C1—O2—C9—C10	−158.66 (16)	C16—C9—C10—C15	70.9 (2)
O2—C9—C16—C21	−7.0 (2)	O2—C9—C10—C11	140.23 (18)
C22—C9—C16—C21	118.0 (2)	C22—C9—C10—C11	23.4 (2)
C10—C9—C16—C21	−118.67 (19)	C16—C9—C10—C11	−103.2 (2)
O2—C9—C16—C17	169.50 (16)	C15—C10—C11—C12	0.2 (3)
C22—C9—C16—C17	−65.5 (2)	C9—C10—C11—C12	174.41 (19)
C10—C9—C16—C17	57.8 (2)	C10—C11—C12—C13	−0.4 (3)
C17—C16—C21—C20	2.6 (3)	C11—C12—C13—C14	0.7 (4)
C9—C16—C21—C20	179.15 (19)	C12—C13—C14—C15	−0.8 (4)
C16—C21—C20—C19	−1.4 (3)	C13—C14—C15—C10	0.6 (3)
C21—C20—C19—C18	−0.3 (4)	C11—C10—C15—C14	−0.4 (3)
C20—C19—C18—C17	0.8 (4)	C9—C10—C15—C14	−174.67 (19)

### Hydrogen-bond geometry (Å, º)

**Table:**

*D*—H···*A*	*D*—H	H···*A*	*D*···*A*	*D*—H···*A*
C14—H14···O1^i^	0.93	2.56	3.444 (3)	158
C21—H21···O2	0.93	2.46	2.810 (3)	103
C5—H5···O1	0.93	2.36	2.701 (3)	101

Symmetry code: (i) −*x*+2, *y*−1/2, −*z*+3/2.
